# Character Decomposition and Transposition of Chinese Compound Words in the Right and Left Visual Fields

**DOI:** 10.1177/2041669516675366

**Published:** 2016-11-04

**Authors:** Hong-Wen Cao, Kai-Fu Yang, Hong-Mei Yan

**Affiliations:** Key Laboratory for Neuroinformation of Ministry of Education, University of Electronic Science and Technology of China, Chengdu, China; Key Laboratory for Neuroinformation of Ministry of Education, University of Electronic Science and Technology of China, Chengdu, China; Center for Information in Biomedicine, University of Electronic Science and Technology of China, Chengdu, China

**Keywords:** Chinese compound words, right visual field superiority, character decomposition, character transposition, character order errors

## Abstract

This study investigated the character decomposition and transposition processes of Chinese two-character compound words (canonical and transposed words) and pseudowords in the right and left visual fields using a dual-target rapid serial visual presentation paradigm. The results confirmed a right visual field superiority for canonical words, but this advantage vanished for transposed words. The findings further indicated that the same quality of lexical processing could be obtained from the foveal and parafoveal regions of the right and left visual fields, regardless of the character order, but not in the periphery of the right visual field. Moreover, the proportion of order reversals peaked at the central position and the shortest exposure time, but it declined with increasing eccentricity and time interval. We concluded that the character transposition of Chinese compound words was significantly sensitive in the periphery of the right visual field. Furthermore, the character order errors were mainly encoded in the foveal vision with a duration of 100 ms, which suggested that the order of the foveally presented Chinese characters was more likely to be reversed at the early stage of visual word processing.

## Introduction

The visual spatial field can be divided into three regions with respect to fixation: foveal (the central 2° of vision), parafoveal (the area between the foveal region and 5° on either side of the fixation), and peripheral (beyond the parafoveal boundary, i.e., beyond 5° from the fixation; Calvo, 2006; Rayner, 2009), which work together to produce our entire visual perception. During reading, eye movements shift a small area of high acuity across the text in the visual spatial field. Researchers posit that visual word identification is a basic process in reading that requires readers to gain useful and detailed information that falls on the fovea or is available in the parafovea, such as to ascertain the identity and the order of the letters in a word (Besner & Humphreys, 1991; Davis, 2010; Inhoff, 1990). Many studies have provided converging evidence that foveal and parafoveal vision play a vital role in normal reading (Lavidor & Walsh, 2004; Rayner, 1998; Rayner & Bertera, 1979). Additionally, the word recognition in central and peripheral vision differs in the extraction speed but not in the quality of lexical processing (Lee, Legge, & Ortiz, 2003).

Previous studies have shown a right visual field advantage for English word recognition (Chiarello, Liu, & Shears, 2001; Dundas, Plaut, & Behrmann, 2013; Ellis, 2004; Simola, Holmqvist, & Lindgren, 2009). More specifically, readers can acquire more information with fixations to the right than to the left (Rayner, 1998). This preference also occurs for Chinese words, there is typically one character to the left and three characters to the right of fixation for the logographic Chinese (Cheng & Yang, 1989; Inhoff & Liu, 1998; Tzeng, Hung, Cotton, & Wang, 1979; Weekes & Bi, 1999). Yet we still know little about the impact of character order encoding of Chinese compound words on the hemispheric asymmetries. Interesting, eye-tracking studies suggested that more information was acquired to the left of fixation when English readers were asked to read from right-to-left (Inhoff, Pollatsek, Posner & Rayner, 1989). Battista and Kalloniatis (2002) posited that this right bias was partly due to the normal reading habit rather than an innate superiority for word recognition of the right visual field.

It must be noted that researchers have devoted considerable effort to exploring how letter identity and letter position are encoded in visual word recognition (Davis & Bowers, 2004; Grainger & Van Heuven, 2003; Schoonbaert & Grainger, 2004). Davis (2003) posited that readers could understand the text with letter transpositions in English, whereas other researchers claimed that words with jumbled letters always carried a transposition cost (Rayner, White, Johnson, & Liversedge, 2006). Some models of visual word recognition have been proposed to explain the presence of effects of letter transposition (Gomez, Ratcliff, & Perea, 2008; Grainger & van Heuven, 2003; Shillcock & Monaghan, 2004; Whitney, 2001). The split-fovea model and SERIOL model have made specific predictions concerning the letter encoding process in the left and right hemispheres (Shillcock & Monaghan, 2004; Whitney, 2001). According to the former model, Monaghan, Shillcock, and McDonald (2004) indicated that the cerebral hemispheres develop different representations of letter order: the individual letters are coded in the left hemisphere, whereas a coarser coding is present in the right hemisphere. The position-specific encoding in the left hemisphere is more sensitive to transpositions (Shillcock & Monaghan, 2004). The latter model posited that encoding the position of letters within words was identical for the left and right hemispheres, whereas the lateral inhibition among adjacent letters would be much stronger in the right hemisphere than in the left hemisphere (Whitney, 2001; Whitney & Lavidor, 2004). Additionally, there are different semantic priming effects in the two hemispheres that are modulated by time interval between the cue and the target, and such effects are stronger in the left input than the right input of the model (Monaghan et al., 2004).

As we noted earlier, it is worthwhile to mention that previous studies primarily explored simultaneously presented whole words rather than sequentially presented characters. To accomplish such investigations, many studies frequently administrate a rapid serial visual presentation (RSVP) which has been established as an effective means of studying the time course of language processing and reading (Potter, 1984). In this paradigm, when participants are asked to identify two targets which are embedded in an RSVP stream of distractors, they can accurately identify the first target (T1); however, the identification of the second target (T2) is severely impaired if it occurs within approximately 500 ms after T1 (Raymond, Shapiro, & Arnell, 1992). This phenomenon is known as attentional blink (AB). Owing to the temporal characteristics of the RSVP paradigm, observers may reverse the temporal order of the two targets, namely T1 is reported as T2, and T2 is reported as T1 (Chun & Potter, 1995; Spalek, Lagroix, Yanko, & Di Lollo, 2012). They can identify the targets but cannot differentiate the order between them because of the loss of episodic distinctiveness. Previous studies demonstrated that the proportion of order reversals for the two targets showed a substantial decrement from Lag 1 (no intervening item) to Lag 3 (two intervening items) during the AB (Bowman & Wyble, 2007; Wyble, Bowman, & Nieuwenstein, 2009). As the stimulus-onset asynchrony (SOA) between the two targets is increased, the temporal discriminability of T1 and T2 increases correspondingly (Wyble et al., 2009). Therefore, the prevalence of order errors is usually used as a measure of temporal competition relationship between the targets. In addition, prior studies had examined the character decomposition and transposition processes of two-character Chinese compound words during the AB task, the results revealed that the AB was eliminated when two Chinese characters form a compound word, regardless of their order (Cao, Gao, & Yan, 2016; Cao, Jin, Li, & Yan, 2014). Nevertheless, it remains unclear how character decomposition and transposition processes of Chinese compound words can modulate visual field asymmetry effects during RSVP reading.

Obviously, efficient language processing might involve generating expectations about upcoming input (Luke & Christianson, 2016; Schuster, Hawelka, Hutzler, Kronbichler, & Richlan, 2016). Chinese readers (Rayner, Juhasz, & Yan, 2005), like English readers (Rayner & Well, 1996), exploit target word predictability during reading. The predictability of a word facilitates word processing is inasmuch as contextually predictable words are more often skipped or fixated shorter than unpredictable words (Balota, Pollatsek, & Rayner, 1985; Kliegl, Grabner, Rolfs, & Engbert, 2004; Kliegl, Nuthmann, & Engbert, 2006). Notably, predictability effects in reading result from graded activation of potentially many words rather than discrete prediction of a specific word (Staub, 2015). Additionally, it is essential to explore the impact of predictability effects on the processing of character decomposition and transposition of Chinese compound words in the right and left visual fields.

The present study intends to explore the hemispheric asymmetries and character order encoding of Chinese two-character compound words and pseudowords using a dual-target RSVP paradigm in which Chinese character pairs of three different categories (canonical words, transposed words, and pseudowords) were presented as stimuli. Specifically, we aim to answer the following two questions: (a) whether the hemispheric asymmetries are affected by the character decomposition and transposition processes of Chinese compound words? Additionally, whether the left–right asymmetries occur during different temporal lags of the two target characters? (b) whether character order errors of canonical words, transposed words, and pseudowords differ in the foveal, parafoveal, and peripheral regions of the right and left visual fields?

## Method

### Participants

Fifty right-handed native Chinese speakers (25 males and 25 females, aged 21–31, *M* = 23.88, *SD* = 1.86) participated in the experiments. All subjects had normal or corrected-to-normal vision in both eyes and were blinded to the purpose of the study. Written informed consent was obtained from the subjects prior to participation. The experiment was approved (approval number: 000101) by the Ethics and Human Participants in Research Committee at the University of Electronic Sciences and Technology of China in Chengdu, China.

### Apparatus

The tasks were performed in a sound-attenuated room that was specially designed for psychophysics experiments, and the room illumination was maintained at the same level for all participants. All stimuli were presented using the Psychophysics Toolbox for MATLAB (Brainard, 1997; Pelli, 1997) running on a high-resolution color monitor (1024 × 1280 pixels, 3 × 8 bit RGB) with a 100-Hz refresh rate.

### Materials

**Figure 1.**
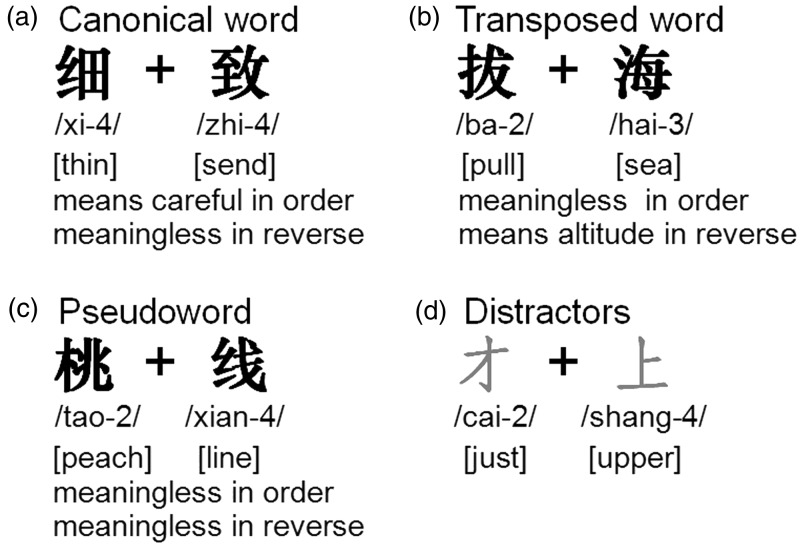
Examples of target words and distractor characters. The Chinese phonetic labeling system (i.e., pinyin) is listed below the character, the number following the pronunciation denotes the character’s tone.

Three categories of word pairs were selected as target stimuli (size 1° × 1°). (a) Canonical words, for example, “细” (thin, T1) and “致” (send, T2), in which the two characters could be integrated into a meaningful word when they are written together in sequence (T1 + T2, meaning careful), but they are meaningless when they are written in the reverse order. (b) Transposed words which were obtained by transposing the order of the constituent characters of its corresponding canonical word. The meaning of a transposed word can be changed (or not) when the order of its component characters is reversed, for example, “拔” (pull, T1) and “海” (sea, T2). However, the two characters cannot form a meaningful word in the order of “拔海” (T1 + T2), but they form a meaningful word in the reverse sequence of “海拔” (T2 + T1, meaning altitude). (c) Pseudoword, for example, “桃” (peach, T1) and “线” (line, T2), in which the phrase is nonsensical regardless of its constituent character order. The target characters were presented in bold, while the distractors were presented in a normal font. The distractors were irrelevant to the targets in terms of their lexical information. Figure 1 illustrates the three types of target words and a distractor.

All chosen pairs of words were high-frequency two-character Chinese compound words. According to the Modern Chinese Frequency Dictionary (1986), the mean frequency was 77.78 (*SD* = 13.76) for canonical words and 79.47 (*SD* = 10.44) for their corresponding transposed words. The mean single-word frequency of the first and second characters were 82.47 (*SD* = 10.76) and 82.18 (*SD* = 10.96) for canonical words, 78.94 (*SD* = 15.23) and 80.68 (*SD* = 10.72) for transposed words, and 76.71 (*SD* = 13.99) and 78.07 (*SD* = 13.85) for pseudowords, respectively. The visual complexity of the compound characters was matched across each stimulus type. The mean number of strokes in the T1 and T2 characters was 9.86 (*SD* = 2.62) and 9.96 (*SD* = 2.33) for canonical words, 10.00 (*SD* = 2.84) and 9.86 (*SD* = 2.32) for transposed words, and 8.86 (*SD* = 2.37) and 8.61 (*SD* = 1.88) for two-character pseudowords, respectively. There were no significant differences in strokes and word frequencies between T1 and T2 among the three conditions (*p* > .05 in all cases). In total, 112 characters of each type were constructed in this study. The distractors included 100 of the most commonly used Chinese characters. The target characters and distractors were randomly presented with the condition that items were presented only once within a trial.

### Procedure

**Figure 2.**
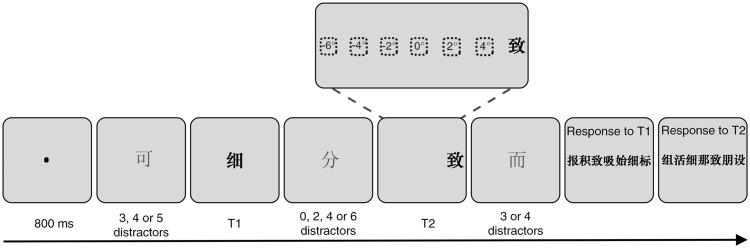
Schematic illustration of a trial sequence. In each trial, the first target character (T1) and distractor characters were always presented at the center of screen (0° eccentricity), and the second target character (T2) was randomly presented at seven different locations (0°, 2°, 4°, and 6° eccentricities in the left and right visual fields). The two target characters were separated randomly by 0, 2, 4, or 6 distractor characters. The presentation duration of each character was 100 ms/item. Note that the characters are depicted larger than their true size (1° × 1°) for the purpose of illustration. In the panel above T2, numbers in the dashed square −6, −4, and −2 represent eccentricities in the left visual hemifield; 6, 4, and 2 represent eccentricities in the right visual hemifield; and 0 represents the fixation point. This scheme shows a trial in which T2 is presented at a 6° eccentricity in the right visual hemifield.

Subjects viewed the stimuli binocularly at a distance of 57 cm from the screen, and their head movements were stabilized in a fixed position by forehead and chin rests during the test. Participants were instructed to maintain fixation throughout each block. Each trial began with a black fixation dot (0.3 in diameter) centered on a grey background, which was extinguished after 800 ms. After this, a RSVP stream of 3, 4, or 5 normal-font Chinese characters (distractors) was sequentially presented at a rate of 100 ms/item. Then, a bold black Chinese character (labeled T1) was presented at the center of the screen (0° eccentricity) and was followed by the appearance of 0, 2, 4, or 6 normal-font Chinese characters, which was systematically varied. Next, an upcoming bold black Chinese character (labeled T2) was presented randomly at different locations centered at 0°, 2°, 4°, and 6° eccentricities in the left and right visual fields. Finally, 3 or 4 normal-font Chinese characters appeared after T2 signaling the end of the stream. All of the distractor characters were presented centrally (0° eccentricity). After the rapid succession, the first panel containing T1, T2, and another five new bold black Chinese characters were presented at the middle of the screen. Participants were asked to give a response to T1 by clicking the mouse on it. Once T1 was chosen, a second panel automatically appeared for participants to identify T2 (Figure 2). Note that the five Chinese characters were chosen from a set of distractors, some of which could be integrated into a meaningful word with either T1 or T2. Subjects were instructed to identify the targets in the order in which they saw them with no wild guesses and to click the blank space on the panel when they did not see the characters.

Participants were given 28 practice trials before the experimental phrase began. Each subject performed four blocks of 84 trials, resulting in a total of 336 individual trials. Each block contained the three types of stimuli pairs, and each category could be presented at seven eccentricities, with four SOAs at each eccentricity. The stimuli types, eccentricities, and SOAs in a given trial were randomly generated by the computer. The order of the blocks was counterbalanced across participants.

## Results

**Figure 3.**
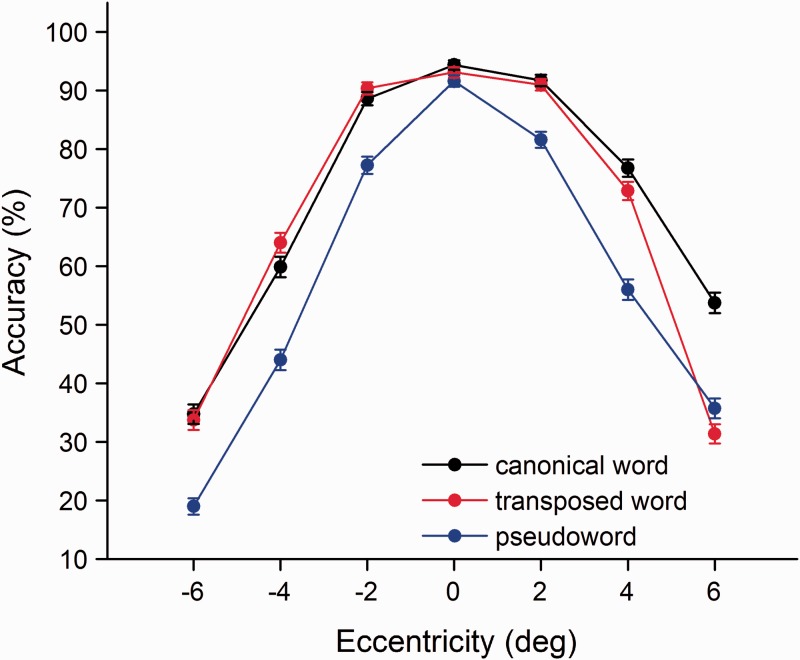
Mean accuracy for the three categories as a function of eccentricity. Eccentricities of −6, −4, and −2 were in the left visual hemifield; eccentricities of 6, 4, and 2 were in the right visual hemifield; and 0 represents the fixation point. Error bars represent the standard error.

The present study aims to investigate the character decomposition and transposition processes of two-character Chinese compound word processing in the right and left visual fields. We mainly analyzed the average accuracy of identification for T2|T1 across seven eccentricities (centered at 0°, 2°, 4°, and 6° eccentricities in the right and left visual fields) and four SOAs (100, 300, 500, and 700 ms) between the two targets under three stimulus categories (canonical words, transposed words, and pseudowords). Targets were counted as correct regardless of the order of identification. Analysis of variance (ANOVA) was computed by subjects (*F*1) and items (*F*2). Table 1 contains the mean percentage of trials for only T1, and only T2 and T2 given the accurate identification of T1 (T2|T1) were accurately identified in the three categories. The average accuracies of T2|T1 under canonical words and transposed words conditions were significant higher than under the pseudowords condition, *F*1(2, 98) = 94.20, *p* < .001; *F*2(2, 670) = 11.81, *p* < .001. It indicated that semantic priming effects facilitated the character decomposition and transposition processes of Chinese two-character compound words in the right and left visual fields. Additionally, the mean accuracy of T2|T1 under the canonical words condition was slightly higher than that under the transposed words condition, *F*1(1, 49) = 11.58, *p* > .05; *F*2(1, 224) = 10.13, *p* > .05 (Figure 3).

**Table 1.** Mean Accuracy in Reporting T1, T2, and T2|T1 in the Three Stimulus Categories During the Dual-Target RSVP Paradigm.

Category type			
	Pseudoword	Transposed word	Canonical word
T1	98.43	98.8	98.82
T2	58.93	69.77	72.07
T2|T1	58.07	68.66	72.07

The four-way (Word type × Visual field × Eccentricity × SOA) ANOVA showed significant main effects of word type, *F*1(2, 98) = 136.98, *p* < .001; *F*2(2, 670) = 36.29, *p* < .001; visual field, *F*1(1, 49) = 115.86, *p* < .001; *F*2(1, 335) = 25.47, *p* < .001; eccentricity, *F*1(6, 294) = 22.84, *p* < .001; *F*2(6, 2010) = 43.08, *p* < .001; and SOA, *F*1(3, 147) = 94.15, *p* < .001; *F*2(3, 1005) = 24.64, *p* < .001. Most importantly, the significant four-way interaction Word type × Visual field × Eccentricity × SOA, *F*1(12, 588) = 13.88, *p* < .001; *F*2(12, 4020) = 3.19, *p* < .01, indicated that character decomposition and transposition processes of Chinese two-character compound words and pseudowords affected temporal processing of visually presented compound words and pseudowords in the left and right visual fields.

**Figure 4.**
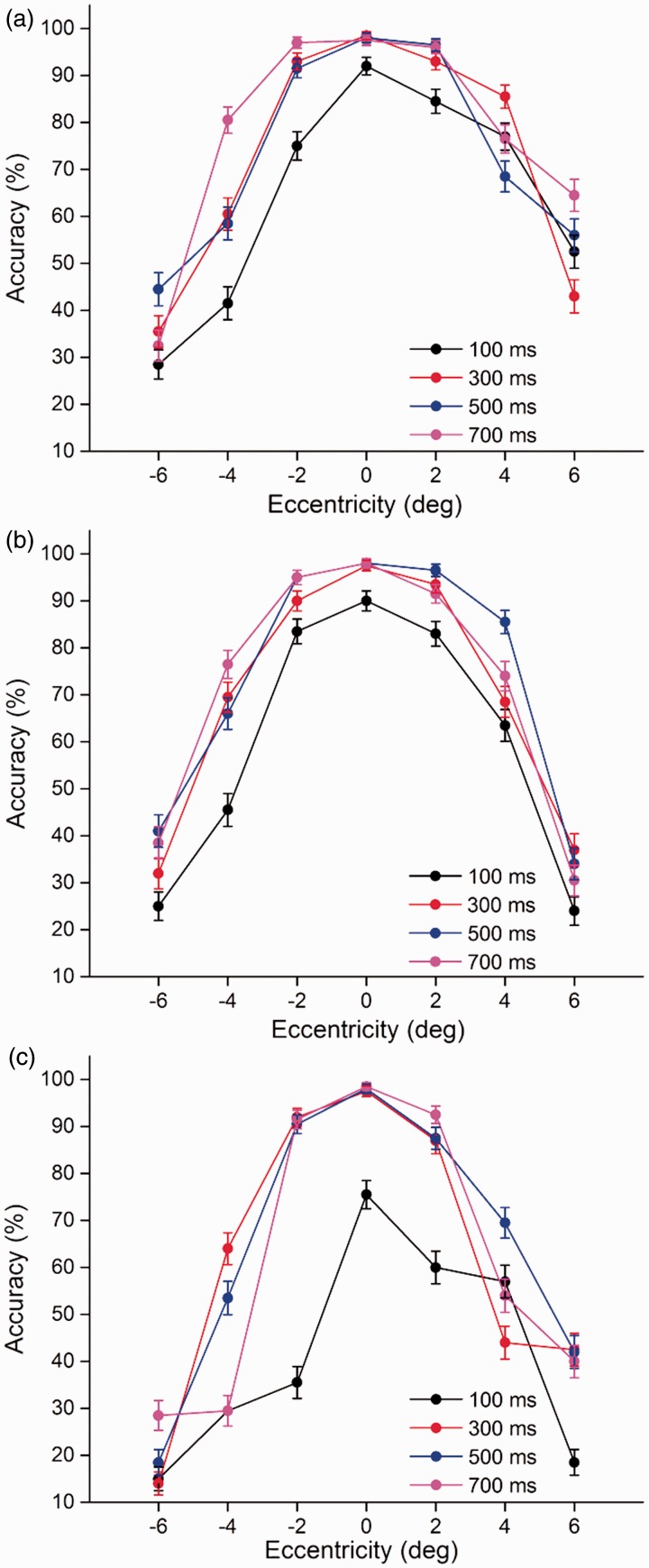
Mean accuracy for the three categories as a function of eccentricity and SOA. Panel a represents the accuracy-eccentricity distribution of canonical words, panel b represents the accuracy-eccentricity distribution of transposed words, and panel c represents the accuracy-eccentricity distribution of pseudowords. Error bars represent the standard error.

To trace the source of the four-way interaction, separate analyses for each visual field were conducted. The characters presented to the right visual field were processed more accurately than those presented in the left visual field for canonical words, *F*1(1, 49) = 34.49, *p* < .001; *F*2(1, 111) = 26.92, *p* < .001, and pseudowords, *F*1(1, 49) = 13.23, *p* < .001; *F*2(1, 111) = 25.45, *p* < .001, and thus revealed right visual advantages for canonical words and pseudowords in the dual-target RSVP task. However, this phenomenon was not found in the transposed word condition, *F*1(1, 49) = 2.20, *p* > .05; *F*2(1, 111) = 1.98, *p* > .05. It suggested that the right visual field superiority for Chinese characters depends on the character order encoding of compound words. In addition, the performance of transposed words at 0°, 2°, and 4° produced a curve almost identical to that in the canonical word condition (paired samples *t* test, all *p* > .05), suggesting we can obtain the same quality of lexical processing between canonical words and transposed words within 4° eccentricity, regardless of the order of the characters within the compound words. There was a significant difference between transposed words and canonical words at the 6° eccentricity, *F*1(1, 49) = 48.25, *p* < .001; *F*2(1, 111) = 46.84, *p* < .001, indicating the character order coding of compound words influenced the extraction of lexical information in peripheral vision (Figure 3). Finally, subsequent analysis revealed a significant difference among the four time intervals, *F*1(3, 147) = 171.36, *p* < .001; *F*2(3, 333) = 34.94, *p* < .001, which indicated that the effect of time interval influenced the identification of the two targets during the sequential presentation. The performance of the two targets was slightly suppressed at a time interval of 100 ms in the canonical word, transposed word, and pseudoword categories compared with the other three SOAs (all *p* < .05), which means that the foveal processing of the first target inhibits the instantaneous central and peripheral processing of the second target with the shortest SOA (Figure 4).

Taken together, the data indicated that the character order encoding within Chinese compound words in right visual field was much more sensitive than that in the left visual field, especially in the periphery of the right visual field.

**Figure 5.**
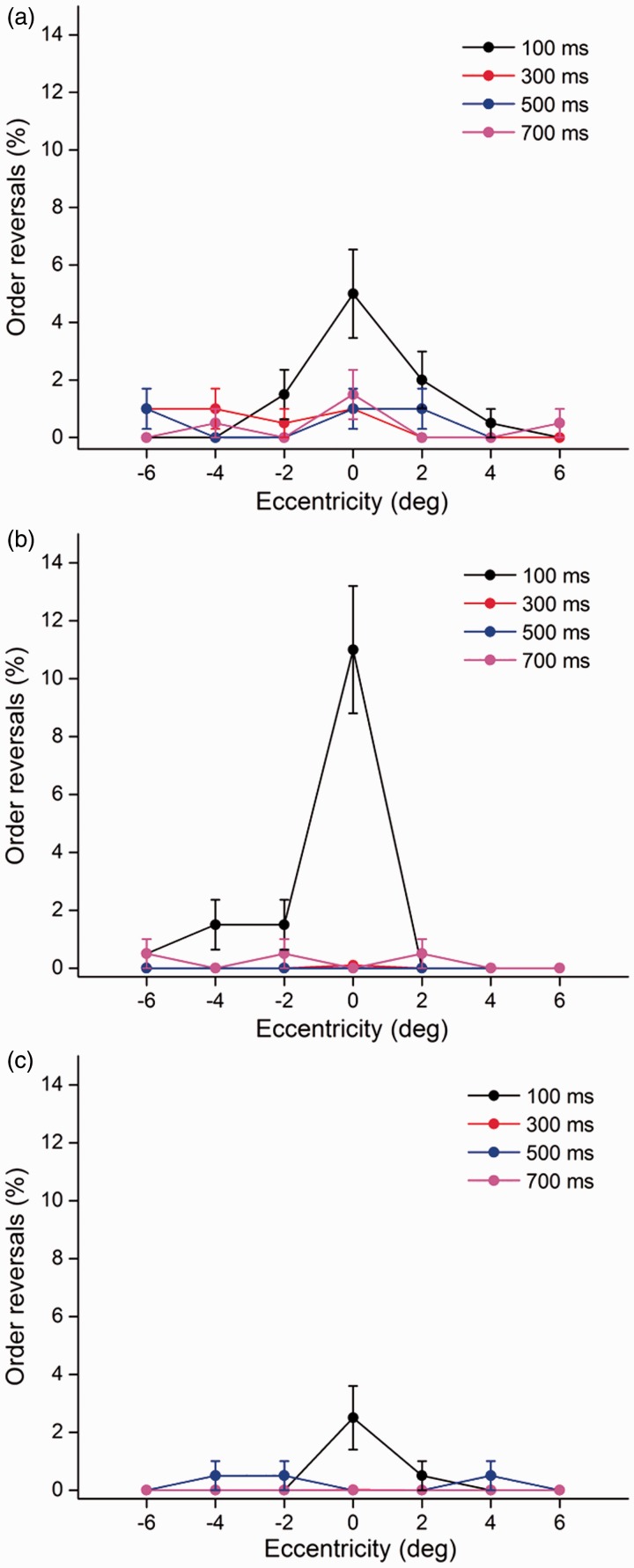
The proportion of order errors for the three categories as a function of eccentricity and SOA. Error bars represent the standard error.

In the RSVP stream, the temporal order of the two targets could be reversed. The proportion of order reversals peaked at centrally (0° eccentricity) presented characters and the shortest exposure (100 ms), but it declined with an increasing eccentricity for all SOAs in the three word categories (Figure 5). The three-way repeated (Word type × Eccentricity × SOA) ANOVA revealed significant effects of word type, *F*1(2, 98) = 8.20, *p* < .001; *F*2(2, 222) = 6.42, *p* < .01; eccentricity, *F*1(6, 294) = 20.69, *p* < .001; *F*2(6, 666) = 26.09, *p* < .001; and SOA, *F*1(3, 147) = 25.06, *p* < .001; *F*2(3, 333) = 26.74, *p* < .001, as well as a three-way interaction among word type, eccentricity, and SOA, *F*1(36, 1764) = 3.81, *p* < .001; *F*2(36, 3996) = 4.86, *p* < .001. Post hoc multiple comparisons revealed significant differences for canonical and transposed words when compared with the pseudowords (all *p* < .01), whereas there were no significant differences between canonical and transposed words (*p* = .583). Moreover, the proportion of order errors at 100 ms is significantly higher than that in the other three SOAs (all *p* < .01). The data demonstrated that the character order errors were mainly encoded in the foveal vision with a duration of 100 ms.

## Discussion

This study investigated the character decomposition and transposition processes of two-character Chinese compound words (canonical and transposed words) and pseudowords in the left and right visual fields. The findings clearly showed that visual word processing varied with character eccentricity and the time interval between the two-character targets in three word conditions. Readers could extract the same quality of lexical processing between canonical and transposed words in the fovea and parafovea, regardless of the order of the characters within compound words. However, character order encoding of words influenced the extraction of lexical information in the peripheral vision. Despite the right visual field superiority for Chinese canonical words, this advantage depended on the character order encoding of compound words. In addition, the order reversals were maximal in the center with the shortest exposure time and almost absent in the far periphery with the longest exposure time. The data demonstrated that the character order errors of Chinese compound words and pseudowords were mainly encoded in the foveal vision with a duration of 100 ms.

One question addressed here is whether the lexical processing of canonical words, transposed words, and pseudowords differs in the right and left visual fields? Previous studies showed that we could extract the same quality of lexical information from the fovea and parafovea (Lee et al., 2003). In this study, the lexical information between canonical words and transposed words in the foveal and parafoveal regions of the right and left visual fields was extracted equally, regardless of the character order within the compound words. However, the performance differed between the two categories in the peripheral region of the right visual field but not in the peripheral region of the left visual field (Figure 3). The character order process influenced the lexical processing of transposed words in the peripheral region of the right visual field. The results might result from the semantic priming between the two constituent characters of the compound words. The first identified character boosted the identification of the second character when the two characters could form a compound word, regardless of their order, and such bidirectional priming was robust in the right and left visual fields, except about transposed words in the peripheral region of the right visual field. More importantly, semantic priming effects of canonical words were stronger in the right visual field than in the left visual field, while such effects were almost identical for transposed words in the right and left visual fields. Additionally, predictability effects were ubiquitous in reading (Luke & Christianson, 2016), and the effects of predictiveness might also interpret the same results, namely the accuracies for two-character compound words were significant higher than that of pseudowords regardless of the order of the constituent characters. Interestingly, the predictability effects of Chinese compound words were more pronounced in the right visual field. Finally, the identification accuracy was slightly suppressed at the time interval of 100 ms in three word categories and was improved with increasing SOAs. The time intervals between the two characters also affected the extraction of lexical processing of words and pseudowords. In sum, our findings support the idea that the lexical information between canonical words and transposed words in the foveal and parafoveal regions of the right and left visual fields (within 4° eccentricity) can be obtained equally is inasmuch as the semantic priming and predictability effect, regardless of the character order. Furthermore, the lexical processing of three word categories was inhibited by the time interval of 100 ms (Figure 4).

The special issue on hemispheric asymmetries is whether the right visual field superiority is affected by the character order encoding of Chinese compound words. Prior studies showed that words were better identified in the right visual field than in the left visual field (Bouma, 1973; Bryden, 1970; Chiarello et al., 2001; Ellis, 2004; Simola et al., 2009). In the present study, the accuracies of T1 and T2 in transposed words were nearly identical in the left and right visual fields, whereas the identification accuracy of canonical words in the right visual field was statistically higher than that in the left visual field, signaling the presence of a right visual field superiority (Figure 3). The absence of a right visual field advantage indicated that this superiority was influenced by the character order encoding of Chinese compound words. We could easily obtain the lexical information of canonical words from the central to peripheral regions of the right visual field, but it was relatively harder to extract the lexical processing of transposed words in the peripheral region of the right visual field. Taken together, the right visual field superiority for Chinese words depends on the character order encoding of compound words, and this superiority is also a consequence of the quality of lexical processing of compound words.

Evidence indicates that the cerebral hemispheres have a different sensitivity when encoding the order of letters within words. The left hemisphere is sensitive to transpositions, which encodes the letter order based on the individual letters (Monaghan et al., 2004; Shillcock & Monaghan, 2004). In addition, the split-fovea model predicts that words with letter transpositions presented in the right visual field may be more perceptually similar to their corresponding base word than that in the left visual field. Our findings are consistent with the predictions of the split-fovea model (Monaghan et al., 2004). As shown in Figures 3 and 4, the accuracies on canonical and transposed words are almost identical in the left visual field, indicating the right hemisphere is relatively insensitive to the character order encoding of Chinese compound words. In contrast, the identification accuracy of transposed words is statistically lower than that of canonical words in the right visual field, suggesting the left hemisphere is more sensitive to the character order encoding of Chinese compound words. Note that the accuracy with transposed words is only statistically lower than that of canonical words in the peripheral region of the right visual field (Figure 3). More specifically, the character transposition within Chinese compound words is mainly sensitive in the periphery of the right visual field.

The last question addressed in this study is whether character order errors of Chinese compound words and pseudowords differ in the fovea, parafovea, and periphery. In the RSVP stream, observers may reverse the temporal order of the two targets. It is worthwhile to ascertain the differences in the transposition probability of Chinese words (canonical words, transposed words, pseudowords) in foveal, parafoveal, and peripheral vision. Note in Figure 5 that the percentage of order errors shows a substantial decrement from the fovea to the parafovea. Specifically, the order confusion for canonical words occurred more often in the foveal and parafoveal regions of the left and right visual fields. Interestingly, the proportion of order errors for transposed words occurred mainly in the foveal and parafoveal regions of the left visual field. We could easily encode the individual character and differentiate the order between them in the right visual field, which provided further evidence that the left hemisphere was more sensitive to the character order coding of Chinese compound words. Moreover, the two characters would be integrated into a single compound word when the second character was presented to the left side of the first fixated character in the transposed word condition. As noted, the perceived order was highly confusable with its actual order in the fovea and at the shortest SOA of 100 ms, indicating the loss of episodic distinctiveness between the two targets. We concluded that the character order errors of Chinese compound words and pseudowords were mainly encoded at the foveal vision with a duration of 100 ms, providing evidence that order of the foveally presented Chinese characters was more likely to be reversed at the early stage of visual word processing. Besides, our results showed that the classical AB effect cannot be observed. In the classical AB paradigm, the two targets are sequentially presented at the same spatial location, while in our dual-target RSVP, T1 is always centrally presented but T2 randomly appears at different spatial positions, which may contribute to the elimination of AB (Figure 4).

## Conclusion

In summary, there was a reliable right visual field superiority with Chinese canonical words and pseudowords but not for transposed words. We could obtain the same quality of lexical processing between canonical words and transposed words in the fovea and parafovea, regardless of the character order within Chinese compound words. However, the character order process influenced the extraction of lexical information from the peripheral vision in transposed words, and more specifically, in the periphery of the right visual field, providing evidence that character transposition process within Chinese compound words was mainly sensitive in the periphery of the right visual field. Finally, the character order errors of the two constituent characters within Chinese compound words were mainly encoded in foveal vision with a duration of 100 ms.
